# Cattle-Derived Unsaturated Aldehydes Repel Biting Midges and Mosquitoes

**DOI:** 10.1007/s10886-021-01347-x

**Published:** 2022-02-02

**Authors:** Elin Isberg, Rickard Ignell

**Affiliations:** Disease Vector Group, Department of Plant Protection Biology, Alnarp, Sweden

**Keywords:** *Culicoides*, *Culicidae*, Host repellent, Electrophysiology, Behaviour

## Abstract

Host-derived repellents offer a novel way to reduce disease vector–host interactions, particularly for vectors and nuisance pests where commercial repellents are not available, e.g., *Culicoides* biting midges. By revising the criteria previously used to identify bioactive volatile organic compounds for *Culicoides nubeculosus*, we identify (*E*)-2-hexenal, (*E*)-2-heptenal, (*E*)-2-octenal, and (*E*)-2-nonenal to be differentially present in the headspace odour of cattle hair and to elicit antennal responses in this research model species. A blend of these unsaturated aldehydes elicited an aversive response in *C. nubeculosus*, as well as a repellent response in three disease vector mosquito species, *Aedes aegypti, Culex quinquefasciatus* and *Anopheles coluzzii*, a response that was stronger than that to the commercially available repellents tested (DEET, IR3535, PMD, icaridin, and *d-*allethrin)*. Culicoides nubeculosus* was behaviourally indifferent to these commercially available repellents tested, except *d-*allethrin to which it was attracted. The identification of a host-derived repellent odour blend, which reduces the interaction between biting midges extends the array of tools to be used in integrated vector management of these and other disease vectors.

**Supplementary Information:**

The online version contains supplementary material available at 10.1007/s10886-021-01347-x.

## Introduction

The biting habit of *Culicoides* biting midges has a dire effect on animal and human welfare, at a global scale. Besides being a nuisance (Kettle 1977), and causing allergic responses in horses (Schaffartzik et al. 2012), biting midges are vectors of emergent vector-borne diseases, including bluetongue and illnesses caused by the Schmallenberg virus, which has changed their epidemiological status in Europe (Purse and Venter 2015). The paucity of knowledge concerning the basic concepts of the biology of biting midges is a lead cause for the lack of efficient methods to control mixed populations of *Culicoides* biting midge species (Harrup et al. 2016). Besides the use of insecticides, other options currently available to control adult disease-transmitting female *Culicoides* biting midges include the use of attractants or repellents (Carpenter et al. 2008). While some progress has been made on the development of attractants, research on repellents, particularly naturally occurring ones, is in its infancy (Harrup et al. 2016; Pickett et al. 2010). As currently available commercial synthetic and natural repellents provide inadequate protection against these ferocious blood feeders (Verhulst et al. 2021), novel research is required to find means to reduce the interaction between these insects and their hosts. Such repellents may also be used against other blood feeders, including mosquitoes.

Non-host volatiles (NHVs) and host-derived repellents, both emanating from potential vertebrate blood hosts, have been demonstrated to deter disease-transmitting insects, including tsetse (Gikonyo et al. 2002; Gikonyo et al. 2003) and cattle flies (Birkett et al. 2004), as well as mosquitoes (Jaleta et al. 2016) under field conditions. In addition, previous studies on *Culicoides* biting midges indicate that both human- (Logan et al. 2009) and cattle-derived (Isberg et al. 2016) volatile compounds act similarly. As such, NHVs should be considered an integral part of integrated pest-management strategies. Non-host volatiles and host-derived repellents are likely indicators of nutritionally, or otherwise, unsuitable hosts (Lyimo and Ferguson 2009). While these deterrents may be species-specific, there are ample examples, not least from herbivorous insects (Cook et al. 2007), but also from hematophagous insects, showing that NHVs and host-derived repellents function when presented at higher than natural doses or outside of the context of the host odour blend (Birkett et al. 2004; Gikonyo et al. 2002; Gikonyo et al. 2003; Jaleta et al. 2016; Logan et al. 2008; Logan et al. 2009).

In this study, we continue our analysis of host-derived repellents against *Culicoides* biting midges (Isberg et al. 2016), through an in-depth chemical and electrophysiological analyses of cattle hair odour. We identify a blend of four unsaturated aldehydes that deter not only *Culicoides* biting midges but also mosquitoes in laboratory and field experiments. A comparative analysis with five repellents used in commercial products, including DEET, ethyl butylacetylaminopropionate (IR3535), icaridin, *p*-menthane-3, 8-diol (PMD), and *d*-allethrin, further reveals the efficacy of this blend. The potential use of the identified repellent odour blend in future management strategies against *Culicoides* biting midges and mosquitoes is discussed.

## Methods and Materials

### Insects

*Culicoides nubeculosus* were provided by the Pirbright Institute, UK, as pupae, and received as adults. Adults were maintained at 27 °C, 65% relative humidity, and at a 12 h: 12 h light: dark cycle, with ad libitum access to water (Isberg et al. 2016). One-to-five-day-old nulliparous, and mated *C. nubeculosus* females (Mair and Blackwell 1996) were used for the electrophysiological and behavioural analyses. Adult *Aedes aegypti, Culex quinquefasciatus,* and *Anopheles coluzzii* were kept under the same conditions, but provided with a 10% sugar solution ad libitum (Majeed et al. 2016). Four-to-six-day-old non-blood-fed female mosquitoes were used for the behavioural analyses.

### Identification of Putative Host-Derived Repellents

Headspace volatile extracts from hair (ca. 15 g) of a group (n = 3) or individual (n = 12) Holstein heifer cattle, cut from their back, neck, and belly, were collected as described by Isberg et al. (2016). In short, the hair was placed in a 500 ml washing bottle (Lenz Laborglas, Wertheim, Germany). The headspace volatiles were collected on an adsorbent column containing 40 mg of Porapak Super Q (PQ; 80/100 mesh; Sigma-Aldrich Chemie GmbH, Steinheim, Germany), by pulling charcoal-filtered air using a modified aquarium pump (Rena 301, Rena France S.A., Meythet, France), from the bottom to the top of the bottle, at 0.1 l min ^-1^ over 24 h, through the column. Prior to collection, the adsorption columns were washed using n-hexane (LabScan, Malmö, Sweden) and pentane (≥99.9, Merck KGaA, Darmstadt, Germany), and eluted with 500 μl of pentane after odour collection. While we acknowledge that this method to collect hair may affect the composition of odorants, we believe this to be rather limited. These volatile extracts were used for electrophysiological and/or chemical analyses.

Bioactive compounds were identified using combined gas chromatography (GC) and electroantennographic detection (EAD) analysis, as well as combined GC and mass spectrometry (MS), using the methodology described by Isberg et al. (2016). The main objective of the GC-EAD and GC-MS analyses was to identify compounds potentially overseen in the study by Isberg et al. (2016), due to the intrinsic problem of low signal-to-noise ratio in the electrophysiological recordings (Isberg et al. 2016; Logan et al. 2009). For this reason, we used a lower criterion for the identification of bioactive compounds; an eluted compound that elicited an EAD response in at least two out of five female *C. nubeculosus*. In total, 30 GC-EAD recordings were conducted on the same number of female biting midges.

Identification of GC-EAD active compounds in the pooled cattle hair headspace volatile extract was done through GC-MS analysis, by comparing their calculated Kováts indices mass spectra to injections of commercially available standards (Table 1), and to reference spectra from custom made and NIST05 (Agilent Technology, Santa Clara, CA, USA) libraries. To quantify the release rate and ratio of the individual bioactive compounds within the grouped and the individual cattle hair samples, 10 ng μl^-1^ of heptyl acetate (99.8% chemical purity; Sigma-Aldrich Chemie GmbH, Steinheim, Germany) was added as an internal standard to a 100 μl aliquot out of the total headspace extract.

**Table 1:** Origin and purity of the synthetic compounds and commercial products used in the current study. The release rate of the unsaturated aldehydes used in the four-component blend and the percentage of the active ingredient (AI) in the commercial products are indicated

Compound	CAS	Purity (%)	Release rate (mg h^-1^)	Product name	Active Ingredient (AI)	CAS	% AI
(*E*)-2-hexenal ^a^	6728–26–3	≥95%	5.6 ± 0.10	Etono ^b^	IR3535	52304–36–6	19
(*E*)-2-heptenal ^a^	18829–55–5	≥95%	5.1 ± 0.09	Sjö&Hav® ^b^	PMD	42822–86–6	20.85
(*E*)-2- octenal ^a^	2548–87–0	≥95%	3.6 ± 0.12	Autan® Protection ^b^	Icaridin	119515–38–7	20
(*E*)-2-nonenal ^a^	1889–56–6	≥95%	2.3 ± 0.07	ThermCell™ ^c^	*d*-allethrin	584–79–2	100
DEET ^a^	134–62–3	97%					

^a^Sigma-Aldrich Chemie GmbH, Steinheim, Germany

^b^Apoteket AB, Solna, Sweden

^c^Thermacell Repellents, Inc., Bedford, MA, USA

### Stimuli

The bioactive compounds identified in the GC-EAD and GC-MS analyses, (*E*)-2-hexenal, (*E*)-2-heptenal, (*E*)-2-octenal, and (*E*)-2-nonenal were used for behavioural analyses (Table 1). In addition, common constituents of commercially available insect repellents, N, N-diethyl-*m*-toluamide (DEET), ethyl butylacetylaminopropionate (IR3535), *p*-menthane-3, 8-diol (PMD), icaridin, and *d*-allethrin, were used either as neat compounds or as part of a commercially available product (Table 1).

### Behavioural Assays

#### *Culicoides nubeculosus*

To assess the behavioural response of *C. nubeculosus* to the putative host-derived repellents and commercially available repellents, a Y-tube olfactometer (Fig. 3a) was used, as described by Isberg et al. (2016). The behavioural assays were performed from 30 min before the onset of the photophase until 90 min after, i.e.*,* at the peak of host-seeking activity (Kettle 1962). To activate and attract the biting midges, synthetic air, containing a metered amount of CO_2_ (600 ppm) and oxygen (20%), balanced by nitrogen (Strandmöllen AB, Ljungby, Sweden), was introduced into both arms, at a rate of 300 ml min^-1^ .

Serial dilutions of a 1:1:1:1 blend, of (*E*)-2-hexenal, (*E*)-2-heptenal, (*E*)-2-octenal, and (*E*)-2-nonenal, reflecting the approximate ratios of compounds in the headspace volatile extract of pooled hair, diluted in hexane (≥97%, Merck KGaA, Darmstadt, Germany), were prepared as stimuli for the assay. In addition, subtractive blends were made, by removing individual components from the full blend, to assess the relative activity of each component, after establishing the most effective aversive dose. The commercial repellents, except *d-*allethrin*,* were similarly diluted in hexane. These stimuli were loaded (10 μl) onto filter papers (1 × 1 cm), and tested against hexane as a control. The solvent was allowed to evaporate for 30 s before the filter papers were attached to steel wires (1.5 cm long) and positioned at the centre of the control and test arms of the Y-tube. *d*-Allethrin was obtained through Thermacell® (Bedford, MA, USA) (Table 1). Commercial pads, containing a known quantity of *d*-allethrin, 0.1 g, were cut into pieces to obtain the required dose, and then heated for 10 min on the Thermacell® heating plate, in order to activate the release of the compound, prior to the introduction of the pads into the treatment arm of the assay. A heated pad, of similar size as the pad used in the treatment, not treated with *d*-allethrin, was used as a control. Stimuli were exchanged in between each experimental replicate, and the position of the treatment was exchanged regularly to avoid bias.

Ten to 15 female *C*. *nubeculosus* were released at the down-wind end of the olfactometer, and then allowed to make a choice for 7 min, where after the position of individual insects in the olfactometer was recorded. Females that entered the treatment arm were considered to be attracted to the stimuli, in combination with CO^2^. In contrast, females that entered the control arm, containing the hexane control and CO^2^, were considered inhibited or deterred by the stimulus. Females that did not make an active choice, i.e., remained in the stem of the Y-tube, were considered non-responders and were excluded from further analysis. It is duly noted that the behaviour displayed by the non-responders could be a consequence of either the stimuli, the CO^2^ added to the assay or both. However, no statistical difference among treatments and dose was found. A total of 20 replicates were made for each stimulus and dose.

#### Mosquitoes

To assess the behavioural response of host-seeking female mosquitoes to the putative host repellent blend and commercially available repellents, a spatial repellency assay (Fig. 4a), modified after Grieco et al. (2005), and developed in concurrence with the World Health Organization (WHO 2013), was used, with minor modification: a Plexiglas® tube (9.5 cm i.d., 30 cm in length), fitted at both ends with trapping cages (10 cm × 9.5 cm i.d.) from the same material as the tube, netted at the far end, and with a netted swing door at the opposite end (Fig. 4a). A metal chamber (15 cm × 10 cm i.d.) (Lindab, Ängelholm, Sweden), housing the stimuli, was fitted onto the ends of the trapping cages, after which both ends were sealed with a plastic lid (Lindab). Through a hole (3 cm i.d.) in each of the plastic lids, air (0.5 l s^-1^) mixed with pulses (0.25 s on and 0.25 s off intervals) of 600 ppm CO_2_, was passed through the bioassay via low-density polyethylene tubing (0.4 cm i.d), using a stimulus controller (SEC-2/b. Syntech, Buchenbach, Germany) according to the protocol described by Majeed et al. (2014). The concentration of CO_2_ was measured through the hole at the centre of the assay (Fig. 3a), using a CO_2_ analyser (LI-820, Licor Bioscience, USA).

Two methods were used to deliver the stimuli. The first method, used for *Ae. aegypti* only, was adapted according to WHO (2013) recommendations, in which 5 ml of a serially diluted (99% ethanol; Solveco AB, Rosersberg, Sweden) stimulus was loaded onto a filter paper (275 cm^2^, Munktell-Ahlström, Helsinki, Finland). The putative host-derived repellent blend, the commercial repellents, and *d*-allethrin were otherwise prepared and delivered as described above. Similarly, an ethanol-treated filter paper was used as a control. The solvent was allowed to evaporate for 15 min after which the filter papers was introduced into the lining of the metal cylinder of either the treatment or control side of the assay. The second method, which was used for all mosquito species, was also based on the recommendations by WHO (2013), in which 100 μl of stimuli, diluted in pentane (Sigma-Aldrich), were applied on a cotton dental roll (0.5 cm i.d., 2 cm in length; 28.2 cm^2^; DAB Dental, Upplands Väsby, Sweden). The solvent was allowed to evaporate for 2 min and then placed in the centre of the metal cylinder at the treatment side, whereas a pentane-only treated dental roll was placed at the control side. Stimuli were exchanged in between each experimental replicate, and the position of the treatment was exchanged regularly to avoid bias.

Twenty female mosquitoes were introduced into the assay through the hole in the centre of the straight tube, and then allowed one min for acclimatisation, after which CO_2_ was added to both ends of the assay. The females were given seven min to make a choice, and then the position of the mosquitoes was recorded. Females entering the trapping cages on either side of the assay was considered to have made a choice. Females that did not make an active choice, hence remained in the central part of the assay, were recorded as non-responders. The behaviour of the mosquitoes was analysed as above. A total of 10 replicates were made for each stimulus and dose.

### Field Evaluation of Putative Host-Derived Repellents

Field evaluation of the blend of putative host-derived volatile compounds was performed at two sites known to contain large populations of biting midges and mosquitoes, Stockhultsgården, 14 km northwest of Markaryd, Sweden (N 56° 32,867′, E 13° 32.542′) and Bjässjön (62°38′16.3”N 17°04′33.9″E), a lake ~30 km northwest of Sundsvall, Sweden. Stockhultsgården has previously been described and used for large field collections of *C. impunctatus* (Isberg et al. 2017). Collections here were performed during June to mid-July, the peak season for blood-feeding *C. impunctatus* in southern Sweden (Isberg et al. 2017). The second site is a lake surrounded by a commercially grown spruce forest and a few recreational residences. The area is known for inhabiting a large population of mosquitoes and biting midges. Collections here were performed from late-July to early August.

Mosquito Magnet ® Liberty Plus traps (Woodstream Co., Brampton, ON, Canada), which combine CO_2_ and the well-characterised host kairomone, 1-octen-3-ol, were used as a source of attraction for host-seeking biting midges and mosquitoes, and served both as a positive control and a platform to assess the efficacy of the host-derived repellent blend. Three pairs of traps per site were used, with each pair separated >150 m, and with the treatment and control traps at each sub-site separated 5 m apart. The components of the blend were released individually to better adjust for differences in their release rate. In addition, 5% butylated hydroxytoluene (≥ 99%, Sigma-Aldrich) was added as an antioxidant. The required release rate (5 mg h^-1^) for each component was achieved by applying 1 ml of neat compound on a cotton dental roll and placing it inside low-density polyethylene sachets (LDPE; 60 × 60 cm) (Rajapack, Göteborg, Sweden) with various film thickness, 0.05 mm for (*E*)-2-heptenal, (*E*)-2-octenal and (*E*)-2-nonenal, and 0.1 mm for (*E*)-2-hexenal. The sachets were weighed directly after the application of the compounds and then once again 24 h after being exposed at 30 °C. Five replicates were carried out simultaneously, and the procedure was repeated twice. The required release rate was achieved by using one or several sachets of the individual compounds (Table 1). The sachets were hung by the odour release point of the Mosquito Magnet™ trap.

Traps were operated for 24 h, after which they were emptied, and sachets exchanged. The trap operating with the treatment was alternated daily to exclude possible position effects. Captured insect were frozen and placed in 70% ethanol for further identification. *Culicoides* biting midges were identified to species using Delecolle (1985), whereas mosquitoes were identified to genus level using MosKeyTool (Gunay et al. 2017), an online database identification key for mosquitoes.

### Statistical Analysis

A *nominal logistic fit model* (JMP® Pro 12.0.1. SAS Institute Inc., Cary, NC, USA) was used to evaluate significant differences in the behavioural responses of female *C. nubeculosus, Ae. aegypti*, *Cx. quinquefasciatus* and *An. coluzzii*. The treatment was used as the dependent variable in the model weighted by a response factor (RF). The RF was calculated as the difference in the number of females choosing the treatment or control arm of the Y-tube assay, or side of the spatial repellency assay, divided by the total number of females making a choice. Dose was set as an independent fixed effect in the model and replicate as a random effect to adjust for the variation in the number of insects introduced into the assay for each of the replicates. The χ^2^ and P value from the likelihood ratio test are reported.

A *restricted maximum likelihood model* (REML) (JMP® Pro 12.0.1. SAS Institute Inc., Cary, NC, USA) was used to identify significant differences in the number of field-caught biting midges and mosquitoes between host-derived repellent treated and control Mosquito Magnet™ traps. Log-transformed numbers of insects caught were added into the model as the response value, to control for over-dispersion in the trap captures. Treatment was added to the model as a fixed effect, together with the different sub-sites. Day, as well as the trap positions, were selected as random effects in the model. Because of the separation between pairs of traps into different sub-sites, these sub-sites were also nested within the position of the traps at the individual sub-sites to adjust for the differences in the data set accounted for by the different environmental factors at the two different test sites. The F and P values from the likelihood effects test are reported.

## Results

### Identification of Tentative Host-Derived Repellents

**Fig. 1:**
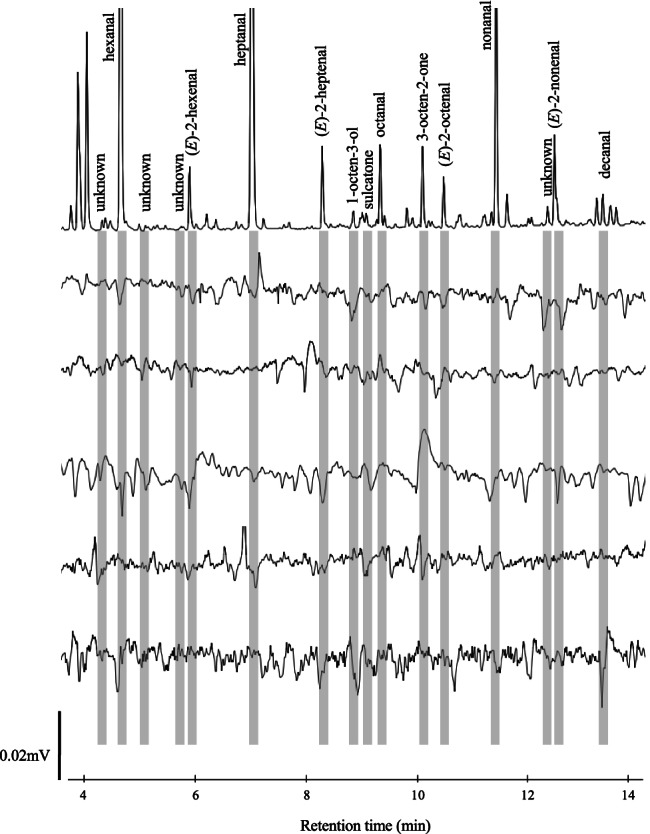
Bioactive compounds in the headspace odour of cattle hair detected by the antennae of *Culicoides nubeculosus*. Combined gas chromatography and electroantennographic detection (GC-EAD) analysis confirms (Isberg et al. 2016) and extends the list of bioactive compounds present in the headspace odour of cattle hair detected by this species of biting midge. Top trace displays the signal of the flame ionisation detector of the GC, whereas the bottom five traces are representative responses obtained from the antenna of individual insects. Bioactive compounds fulfilling previous (Isberg et al. 2016) and the new criteria are indicated in grey

The GC-EAD analysis confirmed and extended the previous study on headspace volatile extraction from cattle hair (Fig. 1; Isberg et al. 2016). Of particular interest was the identification of the unsaturated aldehydes, (*E*)-2-hexenal, (*E*)-2-heptenal, (*E*)-2-octenal, and (*E*)-2-nonenal, which elicited reproducible antennal responses in female *C. nubeculosus*, in at least 2 out of 5 replicates (Fig. 1). Of these, (*E*)-2-nonenal was previously shown to elicit a physiological response in the same species (Isberg et al., 2016). Besides the unsaturated aldehydes, 3-octen-2-one and four unidentified monoterpenes fulfilled the criteria as bioactive compounds using the revised criteria, but were not included in further analysis. 3-Octen-2-one was not considered for further analysis, as we were interested in elucidating the repellent properties of the unsaturated aldehydes, especially as previous field experiments demonstrated that (*E*)-2-nonenal repelled biting midges (Isberg et al. 2017).

**Fig. 2:**
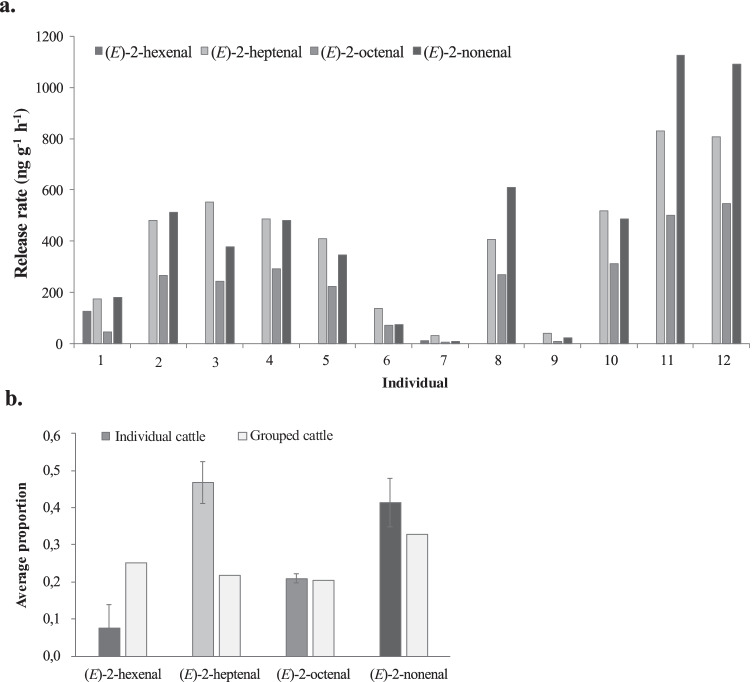
Quantitative differences in the release rate and proportion of unsaturated aldehydes present in cattle hair. **a.** Release of (*E*)-2-hexenal, (*E*)-2-heptenal, (*E*)-2-octenal and (*E*)-2-nonenal from individual Heifer cattle. **b.** Average proportion of the unsaturated aldehydes present in the headspace odour of hair collected from individual cattle or a group of three cattle

Quantification of the unsaturated aldehydes in the headspace volatile extracts of hair from individual heifers revealed variation in 1) the release rate of individual bioactive compounds among heifers and 2) the proportion of compounds released within individual heifers (Fig. 2a, b). While the average proportion of unsaturated aldehydes released from hair of individual heifers varied, the average proportion of unsaturated aldehydes in the headspace volatile extract of pooled hair revealed ratios close to 1:1:1:1 of (*E*)-2-hexenal, (*E*)-2-heptenal, (*E*)-2-octenal and (*E*)-2-nonenal (Fig. 2b). The 1:1:1:1 ratio of the four unsaturated aldehydes was also reflected in the similar release rate of the compounds in the pooled hair sample (Fig. 2a).

### Behavioural Assay

#### *Culicoides nubeculosus*

**Fig. 3:**
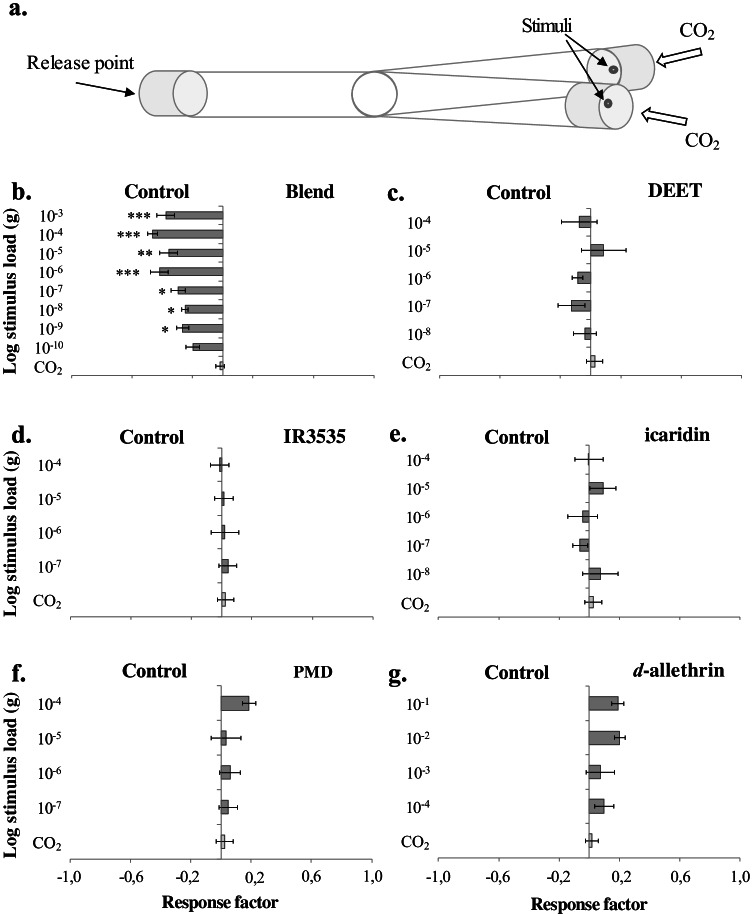
A blend of cattle-derived unsaturated aldehydes uniquely deter host-seeking *Culicoides* biting midges. **a.** Schematic figure of the Y-tube olfactometer used to assess behavioural aversion to the blend of unsaturated aldehydes and commercial repellents. A response factor (±SEM) was calculated to assess the behavioural response of female *C. nubeculosus* to either treatment or control, in which a negative response factor indicates a preference for the control and *vice versa*, using the following treatments: **b.** blend of (*E*)-2-hexenal, (*E*)-2-heptenal, (*E*)-2-octenal and (*E*)-2-nonenal, **c.** DEET, **d.** IR3535, **e.** icaridin, **f.** PMD and **g.** *d*-alletrin (N = 20). Asterisks indicate a significant difference between different concentrations within the different treatments **(b-g)** (* *P* < 0.05, ** *P* < 0.01, *** *P* < 0.001)

The four-component blend elicited a significant behavioural inhibition in female *C. nubeculosus* (χ2 = 22.632, d = 8, *P* = 0.007) (Fig. 3b). Removal of individual compounds from this blend, resulted in an overall significant reduction, or loss of the inhibitory behaviour compared to the full four component blend, when tested at a dose of 10^-6^ g (χ^2^ = 15.442, df = 4, *P* = 0.017) (Supplementary Fig. 1). The five commercially available repellents did not elicit any significant behavioural effect on female *C. nubeculosus* at the tested range of concentrations; DEET (10^-4^–10^-8^ g; χ^2^ = 2.586, df = 5, *P* > 0.05), IR3535 (10^-4^–10^-7^ g; χ^2^ = 1.350, df = 4, *P* > 0.05), icaridin (10^-4^–10^-8^ g; χ^2^ = 2.152, df = 5, *P* > 0.05), PMD (10^-4^–10^-7^ g; χ^2^ = 2.148, df = 4, *P* > 0.05) and d-allethrin (10^-1^–10^-4^ g; χ^2^ = 2.318, df = 4, *P* > 0.05) (Fig. 3c-g).

#### Mosquitoes

**Fig. 4:**
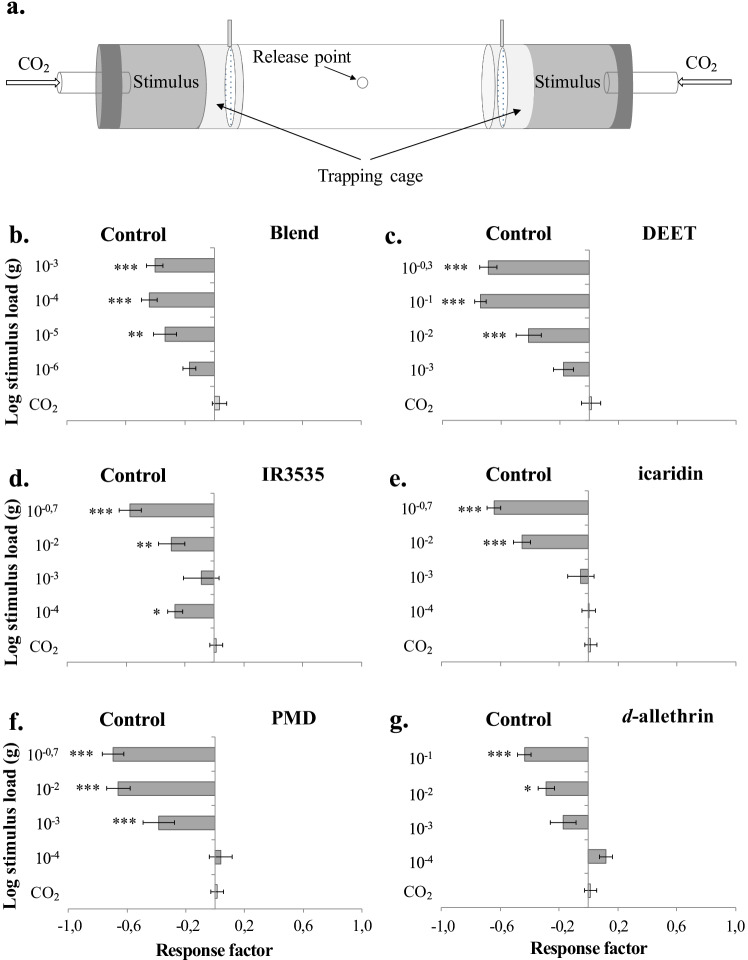
Host-seeking *Aedes aegypti* are repelled by a blend of cattle-derived unsaturated aldehydes and commercial repellents. **a.** Schematic figure of the spatial repellency assay, in which the repellents were dispensed on a filter paper (stimulus). A response factor (±SEM) was calculated to assess the behavioural response of female *Ae. aegypti* to either treatment or control, in which a negative response factor indicates a preference for the control and *vice versa*, using the following treatments: **b.** blend of (*E*)-2-hexenal, (*E*)-2-heptenal, (*E*)-2-octenal and (*E*)-2-nonenal, **c.** DEET, **d.** IR3535, **e.** icaridin, **f.** PMD and **g.** *d*-alletrin (N = 10). Asterisks indicate a significant difference between different concentrations within the different treatments **(b**-**f)** (* *P* < 0.05, ** *P* < 0.01, *** *P* < 0.001)

**Fig. 5:**
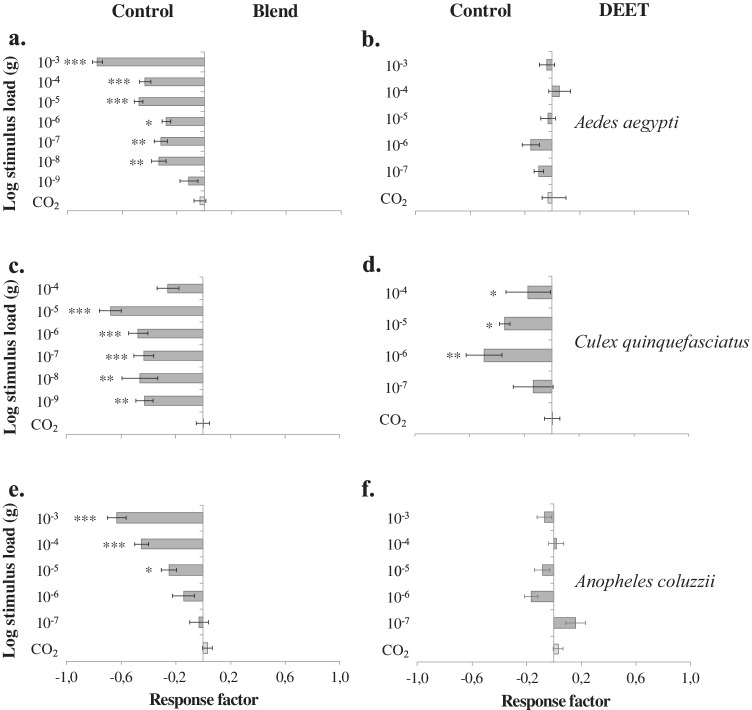
Host-seeking *Aedes aegypti* **(a, b),** *Culex quinquefasciatus* (**c, d)** and *Anopheles coluzzii* (**e, f**) are repelled by a blend of cattle-derived unsaturated aldehydes (**a**, **c**, **e**) and DEET (**b**, **d**, **f**). The repellents were assessed in a spatial repellency assay (see Fig. 4a), with repellents released from cotton wicks placed at either end of the assay (stimulus). A response factor (±SEM) was calculated to assess the behavioural response of female *A. aegypti* to either treatment or control, in which a negative response factor indicates a preference for the control and *vice versa* (N = 10). Asterisks indicate a significant difference between different concentrations within the different treatments **(a**-**f)** (* *P* < 0.05, ** *P* < 0.01, *** *P* < 0.001)

Female *Ae. aegypti* were repelled by a range of concentrations of the four-component blend (10^-3^–10^-5^ g; χ^2^ = 23.818, df = 4, *P* < 0.001), as well as by the commercial repellents tested, when applied on filter paper; DEET (10^-0.3^3–10^-2^ g; χ^2^ = 107.745, df = 4, *P* < 0.001), IR3535 (10^-0.7^, 10^-2^ and 10^-4^ g; χ^2^ = 35.978, df = 5, *P* < 0.001), icaridin (10^-0.7^–10^-2^ g; χ^2^ = 65.478, df = 4, *P* < 0.001), PMD (10^-0.7^–10^-3^ g; χ^2^ = 115.344, df = 5, *P* < 0.001) and d-allethrin (10^-1^–10^-2^ g; χ^2^ = 23.235, df = 4, *P* < 0.001) (Fig. 4). When released from a dental roll, the four-component blend similarly elicited a significant repellent effect in female *Ae. aegypti* (10^-3^–10^-8^ g; χ^2^ = 78.945, df = 7, *P* < 0.001), *Cx. quinquefasciatus* (10^-5^–10^-9^ g; χ^2^ = 29.343, df = 6, *P* < 0.001) and *An. coluzzii* (10^-3^–10^-5^ g; χ^2^ = 39.709, df = 5, *P* < 0.001) (Fig. 5). DEET, when released from a dental roll, elicited a significant behavioural inhibition in *Cx. quinquefasciatus* (10^-4^–10^-6^ g; χ^2^ = 14.172, df = 4, *P* = 0.007), but not in *Ae. aegypti* (χ^2^ = 3.574, df = 6, *P* > 0.05) or in *An. coluzzii* (χ^2^ = 5.246, df = 5, *P* > 0.05) (Fig. 5).

### Field Evaluation of Repellency

#### *Culicoides* spp.

**Fig. 6:**
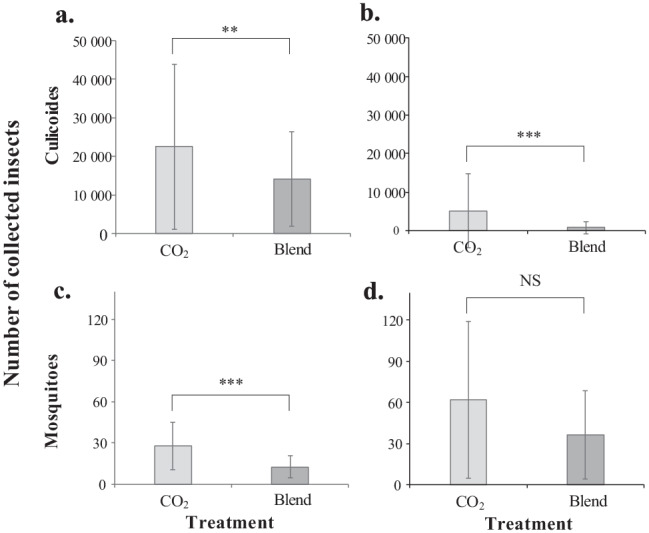
Average number of *Culicoides* biting midges (**a, b**) and mosquitoes (**c, d)** collected at Markaryd (**a**, **c**) and Bjässjö (**b**, **d**) (N = 18 Markaryd, N = 12 Bjässjö, ± SEM). Asterisks indicate a significant difference in the numbers of insects collected between traps treated with the four-component blend of unsaturated aldehydes compared to that of the control traps at the different sites (** *P* < 0.01, *** *P* < 0.001, ns: non-significant)

A total of 657,575 and 71,065 *Culicoides* biting midges were captured in the paired Mosquito MagnetTM traps in Markaryd and Bjässjö, respectively. Most of the biting midges caught in Markaryd (99.94%) and Bjässjö (99.58%), were identified as *Culicoides impunctatus*, whereas lower numbers of species belonging to the Obsoletus complex were caught at each site (Markaryd: 0.05%; Bjässjö: 0.30%). The remaining species caught were collectively categorized as *Culicoides* species (Markaryd: 0.01%; Bjässjö: 0.12%). Traps treated with the four-component blend caught significantly fewer of all *Culicoides* species compared to that of the control traps, at both sites (Markaryd: F = 8.220, df = 1, *P* < 0.001; Bjässjön: F = 17.370, df = 1, *P* < 0.001; Fig. 6a, b). Overall, the average reduction in the number of biting midges captured in the treated traps was 41.6% and 37.6% at Bjässjö and Markaryd, respectively.

#### Mosquitoes

A total of 721 and 1177 from the genus *Aedes*, *Anopheles,* and *Culex* were collected at Markaryd and Bjässjön, respectively. Traps treated with the four-component blend caught significantly fewer mosquitoes at Markaryd (F = 15.282, df = 1, *P* < 0.001), but not at Bjässjön (F = 1.442, df = 1, *P* > 0.05), when compared to the control traps (Fig. 6 cd). Overall, the average reduction in the number of mosquitoes captured in the treated traps was 84.8% and 55.5% at Bjässjö and Markaryd, respectively.

## Discussion

Non-host volatiles and host repellents provide novel means to alleviate problems associated with nuisance and disease-transmitting blood-feeding insects. Unsaturated aldehydes, differentially emitted from the hair of individual heifers, elicited physiological responses in *C. nubeculosus*, as well as behavioural inhibition and repellency of *Culicoides* biting midges and mosquitoes in both laboratory and field experiments, when presented in a blend. As such, the identified host repellent volatiles offer a novel tool to reduce the interaction between biting midges and their potential hosts, and extend the array of tools for integrated vector management of mosquitoes.

Cattle has previously been demonstrated to differ in attraction and in their level of infestation by various fly species (Jensen et al. 2004; Oyarzun et al. 2009), partly due to differences in the volatile components released (Birkett et al. 2004). Similarly, differential attraction of both mosquitoes and *Culicoides* biting midges has been described, and demonstrated to be regulated by the enhanced emission of specific host volatiles (Costantini et al. 2001; Logan et al. 2008; Logan et al. 2009). Differences in volatile profiles among cattle and humans have been linked to differences in their physiological status, where e.g., age, diet and health, and the genetic background of the individual has been shown to influence the susceptibility of the host (Birkett et al. 2004; Elliott-Martin et al. 1997; Haze et al. 2001; Olsson et al. 2014; Shirasu and Touhara 2011; Torr et al. 2006). In this regard, it is interesting to note that of the four unsaturated aldehydes identified in this study, (*E*)-2-nonenal is differentially present in human emanations (Curran et al. 2005) an effect hypothesized by Haze et al. (2001) to be age-dependent. The differential presence of volatile compounds may partly explain the difficulties in identifying *(E*)-2-nonenal and the other unsaturated aldehydes in headspace volatile extracts of cattle (this study, Isberg et al. 2016). While it is unclear whether these signals convey adaptive values, haematophagous insects may decrease their energy expenditure by responding to these type of host volatiles, as observed in herbivorous insects (Lyimo and Ferguson 2009).

The behavioural results obtained for the three mosquito species under laboratory conditions, using an assay designed to assess spatial repellency, suggest that the blend of unsaturated aldehydes provides a direct repellent effect similar to that of the commercial repellents. While the blend of unsaturated aldehydes likely provides a similar effect in *Culicoides* biting midges, we acknowledge that we have not yet provided sufficient support for this hypothesis. Alternatively, the blend of unsaturated aldehydes may ‘mask’ (Deletre et al. 2016) the presence of attractive cues. All of the commercial repellents failed to inhibit the behavioural response of host-seeking *Culicoides* biting midges, with *d-*allethrin even eliciting attraction at the highest dose tested. This is the opposite response to that found for mosquitoes in this study, suggesting that the olfactory system of these dipterans differ considerably in their function. Further analysis is required to address this issue.

The blend of unsaturated aldehydes significantly reduced trap catches of both *Culicoides* biting midges and mosquitoes in the field, thereby providing initial evidence that these cattle-derived volatiles may be used in future management strategies against *Culicoides* biting midges and mosquitoes. Considering the current lack of commercial repellents against *Culicoides* biting midges this is urgently required. While the repellent effect on biting midges was observed at both field sites, a significant effect on mosquitoes was only observed in Markaryd. The apparent lack of a repellent effect at the second field site, was likely due to a change in weather condition, which negatively affected the population of insects at the end of the field trial. Obstacles to overcome in order to develop the blend of unsaturated aldehydes into an effective spatial repellent, and thereby improve the welfare of e.g. cattle, horses, and humans, include a better control of the release of the more volatile constituents in the blend, (*E*)-2-hexenal and (*E*)-2-heptenal, through improved dispenser technology.

## Supplementary Information

Supplementary Fig. 1 Host seeking *Culicoides nubeculosus* are differently repelled by a blend of cattle-derived unsaturated aldehydes and each of the four subtractive blends, in which one component is removed from the four-component blend. A response factor (±SEM) was calculated to assess the behavioural response of female *C. nubeculosus*, in which a negative response factor indicates a preference for the control and *vice versa*. (N = 10). Asterisks indicate a significant difference between different concentrations within the different treatments (* *P* < 0.05, *** *P* < 0.001). (PDF 551 kb)
