# Supplementary figures and images for: Cattle-Derived Unsaturated Aldehydes Repel Biting Midges and Mosquitoes
Source: J Chem Ecol. 2022 Feb 2;48(4):359–69. doi: 10.1007/s10886-021-01347-x (PMC9079034; doi:10.1007/s10886-021-01347-x)

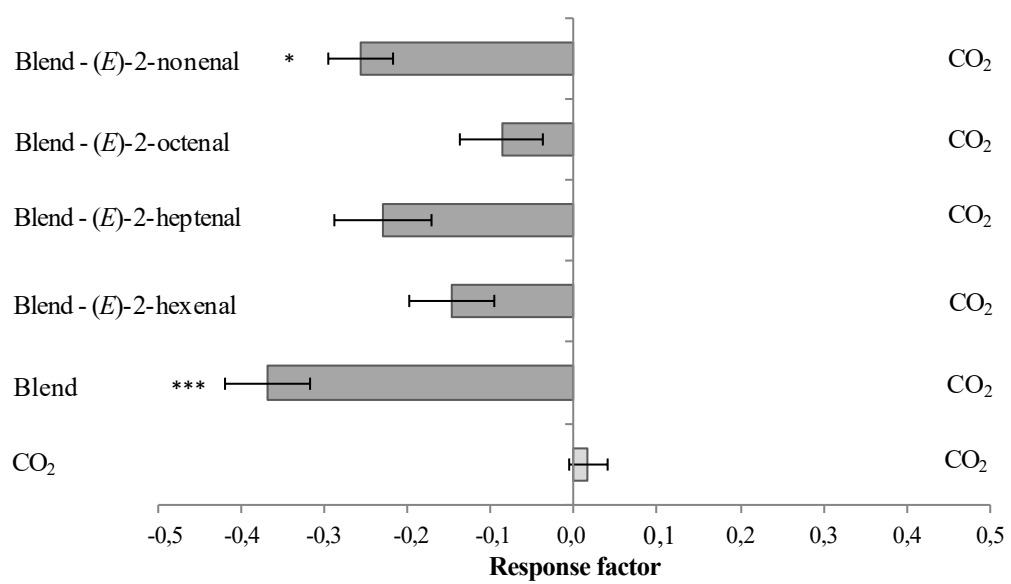

Supplement: Supplementary file 1 — Host seeking Culicoides nubeculosus are differently repelled by a blend of cattle-derived unsaturated aldehydes and each of the four subtractive blends, in which one component is removed from the four-component blend. A response factor (±SEM) was calculated to assess the behavioural response of female C. nubeculosus, in which a negative response factor indicates a preference for the control and vice versa. (N = 10). Asterisks indicate a significant difference between different concentrations within the different treatments (* P < 0.05, *** P < 0.001). (PDF 551 kb) [file 10886_2021_1347_MOESM1_ESM.pdf]
